# Neutrocytopenia associated with a lurasidon therapy in a patient with paranoid schizophrenia

**DOI:** 10.1192/j.eurpsy.2023.2261

**Published:** 2023-07-19

**Authors:** Z. Mielczarek, B. Trędzbor, K. Krysta

**Affiliations:** 1 Students’ Scientific Association, Department of Rehabilitation Psychiatry; 2 Department of Rehabilitation Psychiatry, Medical University of Silesia, Katowice, Poland

## Abstract

**Introduction:**

Lurasidon is a relatively new, second-generation antypsychotic drug with an interesting receptor profile. It is considered safe and has a low risk of side effects. This drug is effecitve in reducing the productive symptoms of schizophrenia, but also has a positive effect on negative symptoms and cognitive functions. It is a medicine with a multi-receptor mechanism of action: it mainly blocks dopaminergic D2 and serotonergic 5-HT2A receptors. According to the Summary of Product Characteristics, adverse reactions such as leucopenia, neutropenia and anaemia were too rare to estimate their frequency.

**Objectives:**

This study describes case of neutrocytopenia and leukocytopenia that are likely to be related to lurasidone and have resolved after discontinuation of this medicinal product. The aim of the article is to highlight the risk of using second-generation antipsychotics in patients and the occurence of blood cells disorders in particular neutrocytopenia.

**Methods:**

An analysis of the medical history records were done to describe the case report.

**Results:**

39-years-old patient treated psychiatrically for 18 years, initially for mood disorders, irritability, behavioural disorders. In 2014 she was diagnosed with paranoid schizophrenia, herlast psychiatric hospitalization took place in 2021. The patient had a history of neutrocytopenia and leukocytopenia. In December 2020, the patient was admitted to the psychiatric ward on account of active aggression against her mother. She was discharged in January 2021 with the recommendation to take paliperidone intramusculary. Due to incomplete remission of psychotic symptoms, the patient received 37 mg of lurasidone since August 2021. The blood count, which was ordered upon admission to the Department of Psychiatric Rehabilitation, showed moderate neutrocytopenia. After discontinuation of lurasidone and the recommended supplementation, the results gradually improved and finally reached the normal range.

**Table:** 

Blood cells type	23.09.2021 r.	04.10.2021 r.	14.10.2021 r.	28.10.2021 r.	10.11.2021 r.	24.11.2021 r.	08.12.2021 r.
Leukocytes	**2,45 x 10^3/μl**	**2,73 x 10^3/μl**	**2,39 x 10^3/μl**	**2,66 x 10^3/μl**	**2,79 x 10^3/μl**	4,09 x 10^3/μl	4,03 x 10^3/μl
Erythrocytes	4,26 x 10^3/μl	4,31 x 10^3/μl	4,48 x 10^3/μl	4,06 x 10^3/μl	4,08 x 10^3/μl	4,39 x 10^3/μl	4,22 x 10^3/μlb
Neutrocytes	**1,04 x 10^3/μl**	**1,60 x 10^3/μl**	**1,24 x 10^3/μl**	**1,53 x 10^3/μl**	**1,78 x 10^3/μl**	2,72 x 10^3/μl	2,62 x 10^3/μl
Lymphocytes	1,05 x 10^3/μl	**0,70 x 10^3/μl**	**0,72 x 10^3/μl**	**0,73 x 10^3/μl**	**0,64 x 10^3/μl**	**0,83 x 10^3/μl**	**0,92 x 10^3/μl**

**Conclusions:**

This case report shows the need for regular monitoring of blood cells parameters in patients treated with second generation antipsychotics, as there is a risk of neutrocytopenia or even agranulocitosis. If there is an obvious correlation, the dose should be reduced or switched to another medicinal product if possible and blood counts should monitored further.

**Disclosure of Interest:**

None Declared

